# Omega-3 Polyunsaturated Fatty Acids as Adjunctive Therapy to Antipsychotic Treatment in Adults with Schizophrenia: A Systematic Review and Meta-Analysis

**DOI:** 10.3390/brainsci16090928

**Published:** 2026-08-31

**Authors:** Aura Patricia Yepes Sarmiento, Thayra Gomez, Kimberly Barrios, Hernan F. Guillen-Burgos

**Affiliations:** 1 Department of Psychiatry, Universidad Simón Bolívar, Barranquilla 080002, Colombia; aura.yepes@unisimon.edu.co (A.P.Y.S.); thayra.gomez@unisimon.edu.co (T.G.); kimberly.barrios@unisimon.edu.co (K.B.); 2 Center for Clinical and Translational Research, Universidad Simón Bolívar, Barranquilla 080002, Colombia; 3 Department of Psychiatry and Mental Health, Pontificia Universidad Javeriana, Bogotá D.C. 110111, Colombia

**Keywords:** antipsychotic agents, dietary supplements, n-3 polyunsaturated fatty acids, randomized controlled trials, schizophrenia

## Abstract

**Highlights:**

**What are the main findings?**

- Pooled analyses found no statistically significant benefit of adjunctive n-3 PUFAs for PANSS total scores, clinical response, all-cause discontinuation, or discontinuation due to adverse events.
- Potential effect modification by baseline n-3 PUFA status, formulation, dose, and concomitant antipsychotic treatment remains unconfirmed.

**What are the implications of the main findings?**

- Routine n-3 PUFA supplementation cannot currently be recommended as adjunctive treatment for schizophrenia based on the available evidence.
- Future trials should standardize EPA and DHA formulations and incorporate membrane biomarkers and prespecified genetic stratification.

**Abstract:**

Background/Objectives: Schizophrenia causes substantial disability and reduced life expectancy, while symptoms frequently persist despite antipsychotic treatment. Long-chain n-3 polyunsaturated fatty acids (n-3 PUFAs) have been proposed as adjunctive therapy because of their anti-inflammatory and neurobiological properties. This systematic review and exploratory meta-analysis evaluated the efficacy and safety of adjunctive n-3 PUFAs in adults with schizophrenia. Methods: The review followed PRISMA 2020 and was prospectively registered in PROSPERO (CRD420251178174). PubMed, Web of Science, Scopus, Ovid, CENTRAL, ClinicalTrials.gov, and ICTRP were searched without language or date restrictions. Randomized controlled trials of n-3 PUFAs added to antipsychotic therapy were included. Risk of bias was assessed using RoB 2. PANSS scores were pooled as mean differences (MDs) using an inverse-variance random-effects model with REML estimation; dichotomous outcomes were pooled as risk ratios (RRs) using an inverse-variance random-effects model with the DerSimonian–Laird estimator or a fixed-effect model with the Mantel–Haenszel method, with a 0.5 continuity correction for zero cells. Results: Of 301 records, 10 randomized controlled trials met the eligibility criteria. Where sufficient comparable data were available, exploratory meta-analyses were conducted. Only one trial provided strict intention-to-treat final PANSS total data. A two-trial sensitivity meta-analysis (116 participants) showed no significant reduction in final PANSS total scores (MD −5.04, 95% CI −14.16 to 4.08; I^2^ = 55.8%). Results were also nonsignificant for clinical response (RR 1.62, 95% CI 0.27–9.78), all-cause discontinuation (RR 1.32, 95% CI 0.31–5.65), and discontinuation due to adverse events (RR 1.35, 95% CI 0.38–4.81). Extreme sensitivity analyses remained nonsignificant. Subgroup signals involving clozapine or baseline fatty-acid status were not consistently replicated. Evidence was limited by few studies, attrition, and substantial heterogeneity in n-3 PUFA formulation, dose, duration, population, and concomitant antipsychotic therapy. Conclusions: Current evidence does not support routine adjunctive n-3 PUFAs for symptom reduction in adults with schizophrenia. The available data cannot identify an optimal EPA/DHA formulation, dose, or responsive clinical subgroup; larger biomarker-informed trials are needed.

## 1. Introduction

Schizophrenia affects approximately 0.5–1% of the population and is associated with a marked reduction in life expectancy [1,2]. Although antipsychotics remain the cornerstone of treatment, their clinical effectiveness is limited in a substantial subgroup of patients. Randomized controlled trials (RCTs) indicate that, after 4 to 6 weeks of treatment, approximately one-third of patients fail to achieve a symptomatic response and nearly two-thirds do not meet remission criteria, indicating that many individuals do not obtain clinically meaningful short-term benefit [3].

In this context, polyunsaturated fatty acids (PUFAs) have gained interest as adjunctive interventions because of their structural and signaling roles in the brain. PUFAs and their bioactive mediators modulate neuronal membrane composition and fluidity, membrane-protein activity, neurotransmitter systems, and immune–neuromodulatory pathways, with established anti-inflammatory and pro-resolving effects [4,5]. Biochemically, n-3 PUFAs derived from α-linolenic acid and n-6 PUFAs derived from linoleic acid share desaturation and elongation enzymes. Competition for these pathways means that substrate availability and the n-6:n-3 balance may influence long-chain PUFA synthesis and membrane incorporation [6,7]. Docosahexaenoic acid (DHA; 22:6 n-3) is particularly important for membrane integrity and synaptic signaling linked to receptor and transporter function [4].

In patients with schizophrenia, alterations in membrane lipid metabolism have been consistently described, including reduced PUFA concentrations in erythrocytes and tissues, particularly DHA (22:6 n-3) and arachidonic acid (20:4 n-6), together with increased oxidative stress [5,8,9]. These findings are consistent with impaired phospholipid homeostasis. On this basis, the membrane phospholipid hypothesis proposes that primary abnormalities in lipid metabolism may contribute to the pathophysiology of schizophrenia and that PUFA supplementation could attenuate symptoms or modify clinical trajectories [10].

Eicosapentaenoic acid (EPA; 20:5 n-3) and DHA (22:6 n-3) can be incorporated into synaptic phospholipids and may influence receptor and ion-channel function by modulating membrane microdomains [4]. They also serve as precursors of lipid mediators involved in the resolution of inflammation and may reduce oxidative injury implicated in schizophrenia [5]. Nevertheless, clinical evidence remains mixed. Recent meta-analyses suggest that n-3 PUFA supplementation, particularly EPA (20:5 n-3) at doses ≥1 g/day, may be associated with modest reductions in symptom severity in selected early-illness or more severely affected populations, but heterogeneity across trials is substantial [11,12]. The field therefore combines biological plausibility with mixed clinical findings.

The 2024 systematic review and meta-analysis by Rossier and Hallahan evaluated n-3 PUFA supplementation across a broader spectrum of psychotic populations and treatment contexts [12]. More recent evidence has also extended investigation of n-3 PUFAs to individuals at ultra-high risk for psychosis, including the PURPOSE randomized clinical trial [13]. These studies highlight the evolving evidence base across different stages of psychosis while also underscoring the need to distinguish populations at risk for psychosis from patients with established schizophrenia. In contrast, the present review applies narrower eligibility criteria, focusing on adults with established schizophrenia receiving n-3 PUFAs as adjunctive treatment to standard antipsychotic therapy. This focused approach addresses the clinical applicability of n-3 PUFA supplementation specifically in established schizophrenia and provides an exploratory quantitative synthesis of outcomes for which sufficiently comparable data were available. Therefore, this systematic review aimed to evaluate the efficacy and safety of n-3 PUFAs as adjunctive therapy to antipsychotic treatment in adults with schizophrenia.

## 2. Materials and Methods

### 2.1. Protocol and Registration

This systematic review was conducted in accordance with the PRISMA 2020 statement [14] and was prospectively registered in PROSPERO (CRD420251178174). The PRISMA 2020 checklist and the complete PubMed/MEDLINE electronic search strategy are provided as Supplementary Materials (File S1 and Table S1).The research question was structured according to the PICOT framework: adults (≥18 years) with schizophrenia diagnosed according to DSM-IV, DSM-5 (including DSM-5-TR), or ICD-10 criteria; n-3 PUFA supplementation, including EPA (20:5 n-3) and/or DHA (22:6 n-3), administered as an adjunct to standard antipsychotic therapy; standard antipsychotic treatment without n-3 PUFA supplementation, with placebo when applicable, as the comparator; and psychiatric symptom severity (PANSS, BPRS, and CGI) and psychosocial functioning or quality of life (GAF and SOFAS) as primary outcomes. Secondary outcomes included discontinuation attributable to supplementation, hospitalization or relapse, adverse events, and mortality. Only RCTs were eligible.

The registered protocol specified a narrative synthesis without formal quantitative pooling. Following peer review and after assessing the availability and comparability of quantitative data, an additional exploratory random-effects meta-analysis was conducted for outcomes with sufficient compatible data. This quantitative synthesis was not prespecified in the registered protocol and was therefore considered post hoc.

The time component was defined as the duration of follow-up reported in each trial. When multiple assessment points were available, end-of-treatment data were prioritized, and interim evaluations were recorded when reported. All eligibility criteria are listed in Table 1.

### 2.2. Information Sources and Search Strategy

The following databases were used to identify records: PubMed/MEDLINE, Web of Science, Scopus, Ovid, and Cochrane CENTRAL, in addition to trial registries (ClinicalTrials.gov). Complementary sources were consulted to identify unpublished or ongoing studies, including the WHO International Clinical Trials Registry Platform (ICTRP) and reference lists of full-text articles. No language or date restrictions were applied.

The search strategy combined MeSH terms and free-text terms related to n-3 PUFAs (e.g., “omega-3 fatty acids,” “EPA,” “DHA,” “docosahexaenoic acid,” “eicosapentaenoic acid,” and “alpha-linolenic acid”) and schizophrenia, together with filters for randomized controlled trials.

### 2.3. Study Selection

Reference management and screening were conducted using Rayyan (Rayyan Web), accessed in late 2025 [15]. Records were exported from each database in RIS format (or PubMed-compatible formats when applicable). Duplicate records were identified and removed using the platform’s automated function, complemented by manual verification.

**Figure 1:**
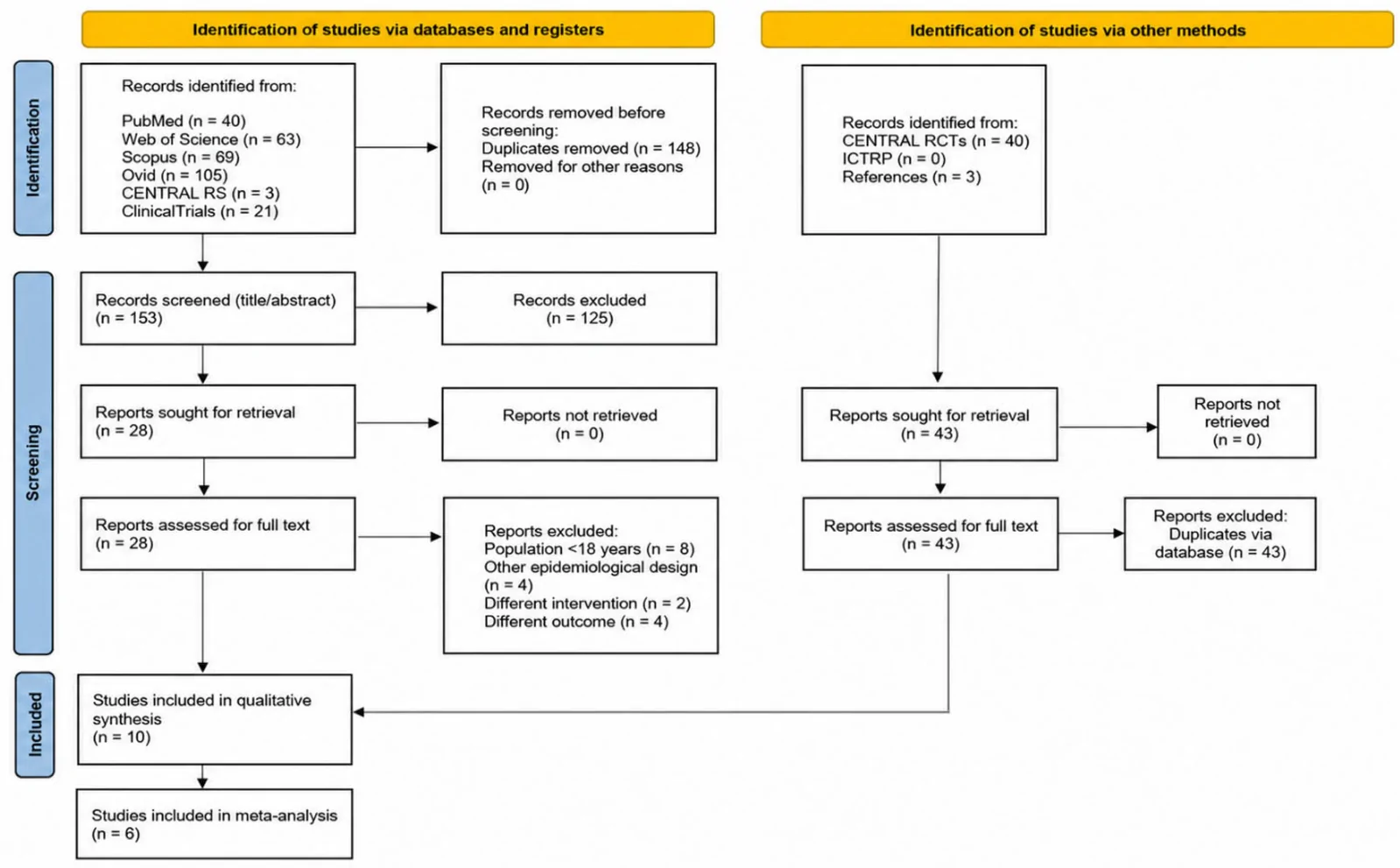
PRISMA 2020 flow diagram illustrating the identification, screening, and selection process of studies included in the systematic review.

Two reviewers (A.Y., T.G.) independently and in a blinded manner screened titles and abstracts, classifying each record as “included,” “excluded,” or “maybe.” Full-text articles were retrieved for records categorized as “included” or “maybe” to determine final eligibility. Discrepancies were resolved through consensus and, when necessary, adjudicated by a third reviewer (HFGB). Reasons for full-text exclusion were documented explicitly and summarized in Table 1. The overall identification, screening, and eligibility process was depicted using the PRISMA 2020 flow diagram in Figure 1.

### 2.4. Data Extraction

Data extraction was performed independently by two reviewers (A.Y. and T.G.) using a standardized form. Extracted study features included country and center, design (parallel-group, factorial, or multi-arm), duration, and assessment schedule. Participant characteristics comprised age, sex, diagnostic criteria, clinical setting, and baseline severity. Intervention characteristics included n-3 PUFA formulation, EPA (20:5 n-3) and DHA (22:6 n-3) composition when reported, daily dose, duration, and route of administration. Comparator characteristics included placebo composition when applicable and concomitant standard treatment.

For continuous outcomes (e.g., PANSS total and subscales and CGI), group-level means and standard deviations were extracted at each time point. When only change scores or repeated-measures/mixed-effects model results were reported, available statistical estimates (e.g., effect estimates, *p*-values, confidence intervals, and interaction time × treatment effects) were extracted for narrative synthesis. For dichotomous outcomes (e.g., discontinuation, adverse events, and mortality), event counts and total sample sizes per arm were recorded.

In trials with multiple active arms (e.g., different n-3 PUFA doses) or factorial designs, the study structure was documented, and analytic strategies were planned to avoid double-counting the control group, using separate dose comparisons or pooling intervention arms when methodologically justified.

Given the clinical and methodological heterogeneity and inconsistent reporting of several outcomes (e.g., BPRS, GAF/SOFAS, hospitalization, or relapse), quantitative synthesis was restricted to outcomes for which at least two clinically and methodologically comparable trials reported sufficient numerical data at the same assessment time. Outcomes that could not be combined were synthesized narratively.

### 2.5. Risk-of-Bias Assessment

Risk of bias was independently assessed by two reviewers (A.Y., T.G.) using the Cochrane Risk of Bias 2 (RoB 2) tool. The following five domains were evaluated: (1) bias arising from the randomization process; (2) bias due to deviations from intended interventions; (3) bias due to missing outcome data; (4) bias in measurement of the outcome; and (5) bias in selection of the reported result. Disagreements were resolved through consensus and, when required, consultation with a third reviewer (HFGB). No automation tools were used during the risk-of-bias assessment.

### 2.6. Statistical Analysis and Data Synthesis

Meta-analyses were conducted when at least two clinically and methodologically comparable trials reported sufficient data for the same outcome and assessment time. Continuous outcomes were summarized using means, standard deviations, and sample sizes. Because all combinable symptom outcomes were measured using the Positive and Negative Syndrome Scale (PANSS), effect estimates were expressed as mean differences (MDs) with 95% confidence intervals (CIs) and pooled using the inverse-variance method. Final scores and changes from baseline were analyzed separately, and lower PANSS scores were considered to favor n-3 PUFA supplementation. The primary analysis of final PANSS total scores was restricted to intention-to-treat data. When fewer than two trials fulfilled this criterion, no pooled estimate was calculated, and the individual trial estimate was reported.

Sensitivity analyses incorporated data from participants who completed follow-up, while an additional, less restrictive sensitivity analysis included trials with substantial attrition. Random-effects models were retained when clinical or methodological heterogeneity precluded the assumption of a common underlying effect. Between-study variance (tau^2^) for continuous outcomes was estimated using restricted maximum likelihood. Wald-type CIs were used in the two-study sensitivity analysis, whereas the Hartung–Knapp adjustment was applied to the additional three-study sensitivity analysis.

Dichotomous outcomes were summarized using event counts and total participants and expressed as risk ratios (RRs) with 95% CIs. Clinical response, all-cause discontinuation, and discontinuation due to adverse events were analyzed separately. A fixed-effect Mantel–Haenszel model was used when I^2^ was below 40% and clinical and methodological comparability supported a common treatment effect. When I^2^ was 40% or higher, or relevant between-study differences were present, an inverse-variance random-effects model with the DerSimonian–Laird estimator for tau^2^ and Wald-type CIs was applied. The Hartung–Knapp adjustment was not used for dichotomous outcomes. When at least one cell within a study contained zero events, a continuity correction of 0.5 was added to all four cells of that study; no correction was applied to studies without zero cells. The Peto method was restricted to the within-trial analysis of mortality in the factorial Bentsen trial and was not used for the principal pooled analyses.

Statistical heterogeneity was assessed using Cochran’s Q test and quantified with the I^2^ statistic. Model selection was not based exclusively on I^2^ because heterogeneity estimates are imprecise when few studies are available. Differences in population characteristics, n-3 PUFA formulation and dose, concomitant antipsychotic treatment, follow-up duration, attrition, and analytical population were also considered. An I^2^ value below 40% was considered potentially unimportant only when clinical and methodological heterogeneity was also limited.

For multi-arm trials, eligible n-3 PUFA groups were combined into a single experimental group by summing sample sizes and event counts for dichotomous outcomes, thereby preventing repeated inclusion of a shared control group. Reports arising from the same trial population were treated as a single study family and were never included simultaneously in the same meta-analysis. Factorial comparisons and subgroup-dependent estimates were analyzed separately when they could not be combined without violating the independence assumption. Outcomes reported by only one eligible trial were presented as individual effect estimates and were not described as meta-analyses.

Two-sided *p*-values below 0.05 were considered statistically significant. Prediction intervals, meta-regression, between-study subgroup analyses, and assessments of small-study effects using funnel plots were not performed because all pooled analyses contained fewer than five trials. Statistical calculations were reproduced in an audited workbook and verified against Cochrane Review Manager algorithms. Outcomes that remained unsuitable for pooling were summarized narratively, and the certainty of the body of evidence was not formally assessed using the GRADE approach.

## 3. Results

### 3.1. Study Selection (PRISMA)

The PRISMA 2020 flow diagram summarizes the identification, screening, and eligibility process (Figure 1). A total of 301 records were identified through database searches. After removal of duplicates and screening of titles and abstracts, 28 full-text articles were assessed for eligibility, of which 10 randomized controlled trials met the inclusion criteria and were included in the qualitative synthesis [16,17,18,19,20,21,22,23,24,25]. Additional records identified through other sources were duplicates of studies already retrieved through database searches and did not contribute to further eligible studies.

### 3.2. Characteristics of Included Studies

The 10 randomized controlled trials were published between 2001 and 2020 and were conducted in China, Slovenia, Norway, Iran, South Africa, the United Kingdom, and the United States [16,17,18,19,20,21,22,23,24,25]. Follow-up ranged from 6 to 16 weeks. Populations included acute inpatients with violent behavior, patients with chronic or persistent symptoms, participants with tardive dyskinesia, and both inpatient and outpatient samples. Concomitant treatment included clozapine, risperidone, haloperidol decanoate, other conventional antipsychotics, and newer atypical antipsychotics.

The interventions were also heterogeneous. Formulations ranged from fish-oil mixtures containing EPA (20:5 n-3) and DHA (22:6 n-3) to purified ethyl-EPA. EPA content typically ranged from 540 mg/day in fish-oil trials to 4 g/day in ethyl-EPA trials; one multi-component essential-PUFA formulation supplied 396 mg/day of EPA together with DHA and α-linolenic acid. Treatment duration ranged from 6 to 16 weeks. Comparators included mineral oil, liquid paraffin, lactose, or low-dose vitamin E placebo preparations. These differences did not permit reliable dose–response or formulation-specific comparisons [16,17,18,19,20,21,22,23,24,25].

Psychiatric symptom severity was most frequently assessed using the PANSS [16,17,18,19,20,21,22,23,24,25]. Additional outcomes included the CGI [17,18,21,25], MOAS [17,18], ESRS [21], and AIMS [20]. Several trials also reported adverse events and other safety outcomes [16,20,21,22,23,25].

Across studies, symptom severity generally decreased over time in both intervention and control groups, with limited evidence of consistent superiority of n-3 PUFA supplementation over placebo [16,17,18,19,20,21,22,23,24,25]. Detailed study characteristics are summarized in Table 2 and Table 3.

### 3.3. Effects of Interventions

Results are presented according to the clinical outcomes assessed across the included studies. A narrative synthesis was used for outcomes with insufficiently comparable data, followed by meta-analysis when at least two clinically and methodologically comparable trials reported the same outcome at a compatible assessment time.

#### 3.3.1. PANSS

In the two Qiao trials involving hospitalized patients with violent behavior, PANSS total and subscale scores decreased in both groups without significant between-group differences. In the 2020 study, the group-by-time interaction was not statistically significant despite reductions at weeks 4 and 8. Similarly, in the 2018 study, PANSS scores decreased at weeks 4, 8, and 12, but n-3 PUFA supplementation was not superior to placebo (*p* > 0.7 for all between-group comparisons) [17,18]. Bošković et al., in a four-arm trial of placebo, vitamin E, essential PUFAs, and their combination, also found no treatment effect on PANSS total or subscale scores after 4 months [19].

Bentsen et al. reported that treatment effects differed according to baseline erythrocyte PUFA status. Among patients with low baseline PUFA levels, EPA (20:5 n-3) monotherapy was associated with a less favorable PANSS total course than placebo (+12.6 points; 95% CI 1.0–24.4; *p* = 0.03), whereas concomitant vitamins E and C attenuated this effect. No differences were observed among patients with high baseline PUFA levels [16]. In the 6-week trial by Manteghiy et al., n-3 PUFAs were added to risperidone; apart from a single difference in general psychopathology at week 3, no consistent between-group differences were observed [20]. Emsley et al. likewise found no difference between EPA (20:5 n-3) and placebo in baseline-to-endpoint PANSS change (*p* = 0.9) [21].

In Peet et al., ethyl-EPA (20:5 n-3) at 2 g/day was superior to placebo for PANSS total (*p* = 0.04) and general psychopathology (*p* = 0.03), without differences in positive or negative subscales; the 1 g/day and 4 g/day arms did not show overall superiority [22]. In Emsley et al., the percentage change in PANSS total at 12 weeks favored ethyl-EPA (12.6 ± 14.0 vs. 3.1 ± 13.3; *p* = 0.03), although the difference was attenuated after adjustment for changes in dyskinesia (*p* = 0.09) [23]. A pilot trial by Peet et al. suggested a greater percentage improvement in PANSS total with EPA (20:5 n-3) than with DHA (22:6 n-3) or placebo [24]. Fenton et al. found improvement over time in both groups without a significant group-by-time interaction [25].

#### 3.3.2. CGI

Qiao et al. assessed CGI–Severity and reported improvement at weeks 4, 8, and 12 relative to baseline, without between-group differences [18]. Fenton et al. reported identical CGI values at week 16 (3.5 ± 0.7 in both groups), with a time effect but no group-by-time interaction [25]. Emsley et al. collected CGI assessments for tardive dyskinesia and did not report a differential effect of EPA (20:5 n-3) on the global clinical impression of psychotic symptoms [21].

#### 3.3.3. Secondary Outcomes Related to Adverse Events

Bentsen et al. assessed adverse events using the UKU scale and found no association between EPA (20:5 n-3) and/or vitamin allocation and the total number of adverse events. Nine serious adverse events were recorded in the intention-to-treat population; although two deaths occurred in EPA-containing groups, mortality was not associated with treatment allocation (Fisher’s exact *p* > 0.48) [16]. Peet et al. reported similar rates of new adverse events across groups (placebo, 52%; 1 g, 50%; 2 g, 34%; and 4 g, 59%), predominantly mild gastrointestinal symptoms [22]. In Fenton et al., upper respiratory infection and diarrhea were the most common adverse events in the EPA group (19% each) [25]. Manteghiy et al. reported mild extrapyramidal and gastrointestinal effects without group-specific data or discontinuations due to adverse events [20]. In Emsley et al., one participant withdrew after an overdose; no between-group differences were observed in parkinsonism, dystonia, or akathisia, while dyskinesia improved more with ethyl-EPA at 12 weeks (t = 2.82; *p* = 0.008) [23].

#### 3.3.4. Mortality

Only Bentsen et al. reported deaths during follow-up (two deaths in EPA-containing groups), with no statistical association between mortality and treatment allocation [16].

### 3.4. Meta-Analysis Results

Four outcome domains were quantitatively evaluated, yielding six pooled estimates, including sensitivity analyses. None showed a statistically significant difference between adjunctive n-3 PUFAs and control interventions.

Only Fenton et al. (2001) [25] reported final PANSS total scores for a strict intention-to-treat population; therefore, the primary analysis could not be pooled. This trial (*n* = 87) found no significant difference between n-3 PUFA supplementation and placebo (MD −1.00, 95% CI −8.15 to 6.15; *p* = 0.784). A sensitivity analysis of Fenton et al. (2001) [25] and Peet et al. (2001) [24], including 116 participants, also found no significant improvement in final PANSS total scores (MD −5.04, 95% CI −14.16 to 4.08; *p* = 0.279; I^2^ = 55.8%). After adding Qiao et al. (2018) [18] in an extreme sensitivity analysis, the result remained nonsignificant (three trials; 142 participants; MD −4.06, 95% CI −17.26 to 9.14; *p* = 0.317; I^2^ = 16.4%). Change-from-baseline scores could not be pooled; however, Emsley et al. (2002) [23] individually reported a significant effect favoring n-3 PUFA supplementation (MD −9.50, 95% CI −17.96 to −1.04; *p* = 0.028).

Clinical response was evaluated in two trials involving 106 participants [21,24]. Response occurred in 8 of 54 participants receiving n-3 PUFAs and 4 of 52 controls, with no significant between-group difference (RR 1.62, 95% CI 0.27 to 9.78; *p* = 0.601; I^2^ = 46.1%).

All-cause discontinuation was assessed in three trials involving 246 participants [21,22,23]. Discontinuation occurred in 21 of 153 participants receiving n-3 PUFAs and 15 of 93 controls, with no significant between-group difference (RR 1.32, 95% CI 0.31 to 5.65; *p* = 0.708; I^2^ = 49.9%). The extreme sensitivity analysis, which added Qiao et al. (2018) [18], was also nonsignificant (RR 0.83, 95% CI 0.53 to 1.29; *p* = 0.407; I^2^ = 34.4%).

Discontinuation due to adverse events was reported in three trials [21,22,23], occurring in 9 of 153 participants receiving n-3 PUFAs and 3 of 93 controls. No significant difference was observed (RR 1.35, 95% CI 0.38 to 4.81; *p* = 0.643; I^2^ = 11.2%). Other safety outcomes, including overall adverse events, serious adverse events, and mortality, could not be pooled because comparable data were insufficient.

### 3.5. Quality Assessment for Risk of Bias

Qiao et al. (2020), Qiao et al. (2018), Bošković et al. (2016), and Emsley et al. (2006) were judged to be at high risk of bias [17,18,19,21]. In all four studies, Domain 3 (missing outcome data) was the primary driver because of high attrition (30–50%) or between-group imbalances (e.g., 19% vs. 33%), without robust imputation or sensitivity analyses and with a plausible likelihood that missingness depended on the true outcome value.

Some concerns were assigned to Bentsen et al. (2013) (low risk in Domains 1, 2, and 4; concerns in Domain 3 because of the pattern and proportion of missing data at 16 weeks) [16]; Manteghiy et al. (2008) (allocation concealment not described, insufficient reporting of missing data, and no protocol or statistical analysis plan) [20]; Emsley et al. (2002) (randomization method not described) [23]; Peet et al. (2002) (limited information in Domains 1, 4, and 5) [22]; and Fenton et al. (2001) (incomplete information in Domains 1, 3, and 5, with moderate missingness) [25].

**Figure 2:**
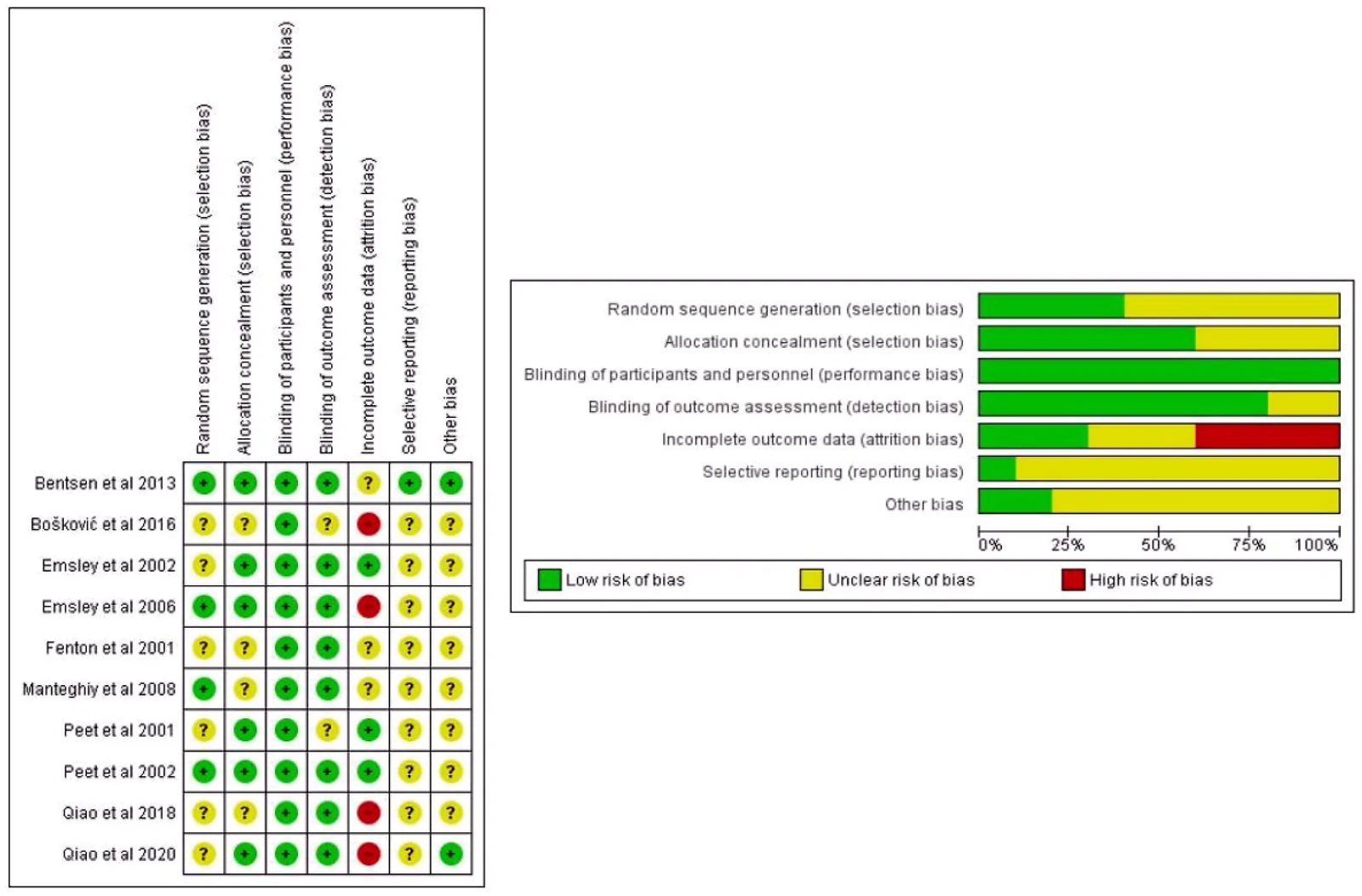
Risk-of-bias assessment of the included randomized controlled trials using the Cochrane RoB 2 tool [16,17,18,19,20,21,22,23,24,25].

The study by Peet et al. (2001) was judged to be at low risk of bias because sequence generation and allocation concealment were managed by an independent third party, blinding was maintained, adherence was high, intention-to-treat analysis was used, outcome measurement was valid, and reported outcomes were consistent with the described plan [24] (Figure 2).

**Figure 3:**
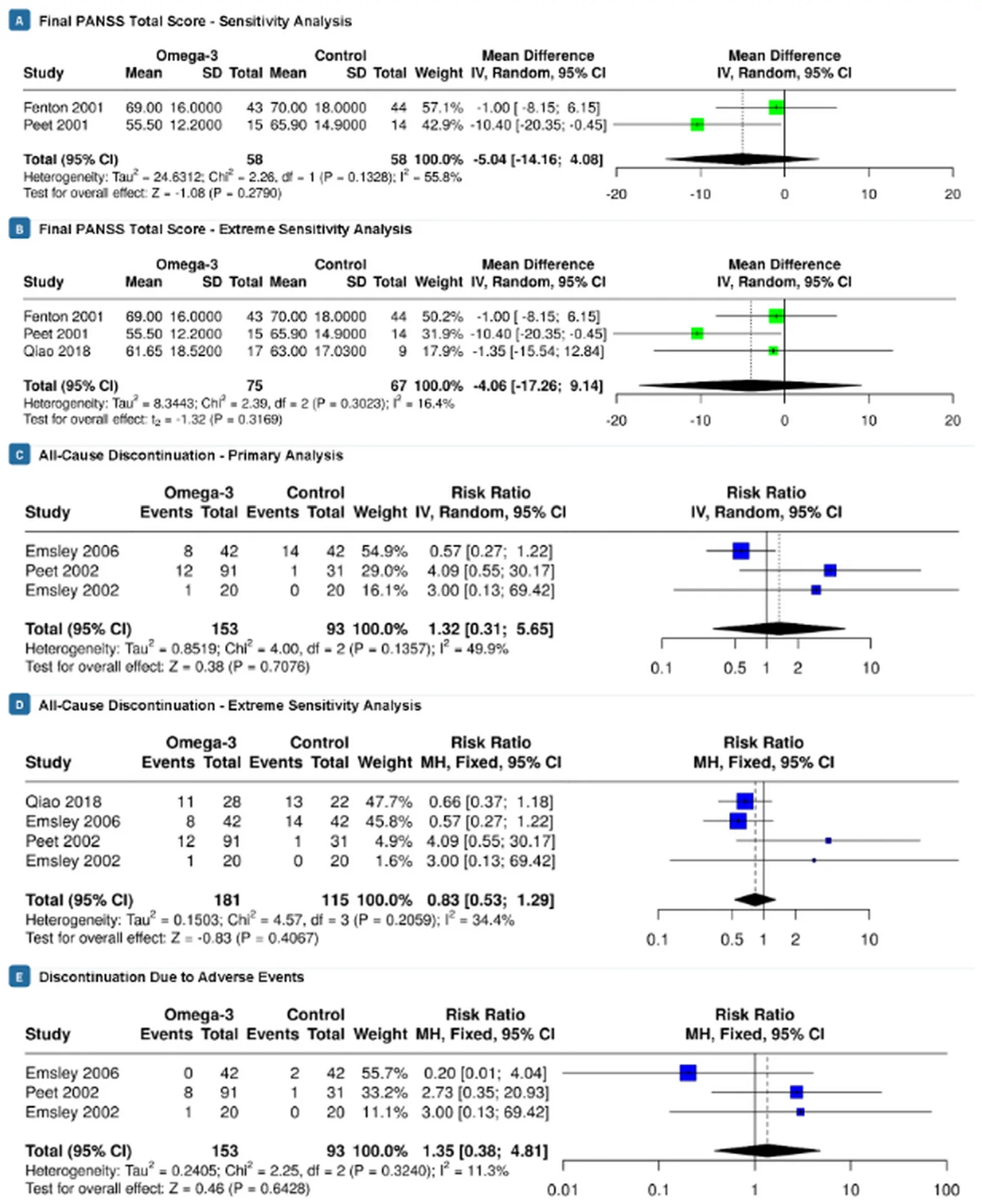
Forest plots of selected exploratory quantitative syntheses of adjunctive n-3 polyunsaturated fatty acids versus control interventions in adults with schizophrenia [18,21,22,23,24,25]: (**A**) final PANSS total score, sensitivity analysis; (**B**) final PANSS total score, extreme sensitivity analysis; (**C**) all-cause discontinuation, primary analysis; (**D**) all-cause discontinuation, extreme sensitivity analysis; and (**E**) discontinuation due to adverse events.

Selected forest plots from the exploratory quantitative syntheses are presented in Figure 3.

## 4. Discussion

Across the pooled analyses, adjunctive n-3 PUFAs did not demonstrate statistically significant benefits for final PANSS total scores, clinical response, all-cause discontinuation, or discontinuation due to adverse events, and sensitivity analyses did not alter this conclusion. Narrative evidence likewise showed no consistent benefit for PANSS or CGI outcomes. Evidence for hospitalization, relapse, mortality, and functional outcomes remained sparse. Short-term tolerability was generally comparable to placebo and was characterized mainly by mild gastrointestinal adverse events. Isolated subgroup signals involving clozapine or baseline PUFA status were not consistently replicated [16,22]. Given the small number of trials and wide confidence intervals, these nonsignificant findings should not be interpreted as proof of equivalence.

A major source of uncertainty was intervention heterogeneity. The trials evaluated mixed fish-oil products and purified ethyl-EPA, with materially different EPA (20:5 n-3) and DHA (22:6 n-3) content, EPA doses, placebo oils, and treatment durations. Consequently, the pooled estimates represent average effects across regimens that are not necessarily biologically interchangeable. The available data cannot establish a dose–response relationship or determine whether EPA-only and EPA/DHA formulations have different clinical effects.

Clinical heterogeneity was also substantial. Participants ranged from acutely hospitalized patients with violent behavior to outpatients with chronic persistent symptoms or tardive dyskinesia, and concomitant treatment included clozapine, risperidone, haloperidol decanoate, and other conventional or atypical antipsychotics. Differences in stage and duration of illness, baseline symptom severity, clinical setting, and antipsychotic exposure may have modified treatment effects and probably contributed to between-study inconsistency. The small number of trials precluded credible subgroup meta-analysis, so the apparent clozapine signal remains exploratory.

Biomarker assessment was insufficient to distinguish pharmacological inefficacy from inadequate biological exposure. Only the two Qiao trials measured plasma EPA (20:5 n-3) and DHA (22:6 n-3) longitudinally [17,18], while Peet et al. assessed erythrocyte membrane fatty acids only in a clozapine subgroup [22]. Most trials therefore could not determine whether participants were n-3 PUFA deficient at baseline, achieved meaningful membrane enrichment, or adhered biologically to supplementation. Moreover, n-3 and n-6 PUFAs share desaturation and elongation pathways; competition between their substrates may influence long-chain PUFA synthesis, membrane incorporation, and the balance of derived lipid mediators [6,7]. Future trials should measure baseline and post-treatment plasma and erythrocyte EPA, DHA, arachidonic acid (20:4 n-6), and n-6:n-3 ratios, together with dietary intake and adherence.

Genetic variation in lipid metabolism is another plausible source of heterogeneity. Nadalin et al. found that variants in *PLA2G4A* and *PLA2G6* were associated with changes in PANSS psychopathology and/or metabolic parameters during antipsychotic treatment, whereas *PLA2G4C* was not associated with treatment response [26]. Subsequent studies reported that the PPARα L162V variant was associated with LDL-cholesterol changes in a risperidone/paliperidone subgroup [27] and that the COX-2 (*PTGS2*) rs689466 variant was associated with HDL-cholesterol changes among clozapine-treated patients [28]. These studies evaluated response to antipsychotic medication, not to n-3 PUFA supplementation; their relevance to the present intervention is therefore indirect and hypothesis-generating. Nevertheless, they support prespecified investigation of lipid-metabolism genotypes as potential modifiers in future biomarker-stratified n-3 PUFA trials.

These findings should also be interpreted in light of the risk of bias. Several trials that were at high risk of bias because of incomplete outcome data reported improvement in both groups without significant between-group differences [17,18,19,21]. Trials with some methodological concerns likewise produced mixed findings [20,25]. A few small studies suggested a benefit of ethyl-EPA, but one apparent PANSS improvement was attenuated after adjustment for changes in dyskinesia [21,23,24]. The only trial judged to be at low risk of bias reported benefit confined to a clozapine subgroup [22]. Adverse events were generally mild and comparable to placebo, most commonly gastrointestinal or extrapyramidal symptoms [16,20,21,22,23,25]. Mortality was rarely reported and was not associated with treatment allocation [16]. Overall, isolated positive results should be interpreted cautiously because of imprecision, risk of bias, and clinical heterogeneity.

These findings are broadly consistent with previous reviews. Fusar-Poli et al. found no significant effect of EPA (20:5 n-3) as adjunctive treatment in established schizophrenia [29]. Sommer et al. ranked fatty acids behind other adjunctive anti-inflammatory strategies [30], suggesting that any benefit is likely modest or context-dependent. Cho et al. emphasized substantial heterogeneity and identified isolated positive findings, particularly among patients receiving clozapine, alongside several unfavorable trials [31]. Bozzatello et al. proposed that n-3 PUFAs may be more promising in early illness, first-episode psychosis, or clinical high-risk states; however, evidence in established schizophrenia remained heterogeneous [32]. The Cochrane review by Irving et al. similarly concluded that the evidence remained inconclusive and that the use of n-3 PUFAs for schizophrenia remained experimental [33].

The main strengths of this review include a comprehensive and transparent search strategy, duplicate study selection and data extraction, qualitative and quantitative synthesis, and formal assessment of risk of bias using RoB 2. The review also incorporated the eligible randomized trials and explicitly considered heterogeneity in formulation, dose, population, baseline PUFA status, and concomitant antipsychotic treatment.

### Limitations and Future Directions

The evidence base was limited by substantial clinical and intervention heterogeneity, high attrition, incomplete reporting of randomization and allocation concealment, few registered protocols, short follow-up, and sparse reporting of functional or hard clinical outcomes. Inconsistent measurement of baseline and achieved n-3 PUFA status further limited interpretation of biological exposure and potential effect modification.

The quantitative synthesis also had important limitations. Each meta-analysis included only two to four trials, reducing precision and the reliability of heterogeneity estimates, and the primary final-PANSS analysis could not be pooled because only one trial provided strict intention-to-treat data. Publication bias, prediction intervals, meta-regression, and between-study subgroup analyses could not be assessed because every pooled analysis contained fewer than five trials. The certainty of evidence was not formally evaluated using GRADE. An additional limitation is that the quantitative synthesis was not prespecified in the registered protocol and was conducted post hoc after assessing the availability and comparability of quantitative data. Accordingly, the pooled estimates should be considered exploratory and interpreted cautiously.

From a clinical perspective, n-3 PUFAs should not be routinely added to antipsychotic therapy solely to reduce psychotic symptoms. The evidence does not identify an effective EPA/DHA formulation, dose, treatment duration, or clinical subgroup, including patients receiving clozapine. Use for other clinical indications should remain individualized and must not replace evidence-based antipsychotic treatment.

Future RCTs should also prespecify treatment duration and standardized assessment time points to improve comparability across studies. For future trials, an acute-treatment period of approximately 12 weeks could provide a pragmatic window for evaluating symptomatic efficacy, while longer follow-up of 6–12 months would allow assessment of relapse, hospitalization, functioning, quality of life, and longer-term safety. Core clinical outcomes should be assessed using validated and consistently reported instruments, including the PANSS for psychotic symptoms and standardized measures of global clinical status, functioning, and quality of life. To facilitate future quantitative synthesis, trials should prespecify outcome definitions and assessment time points and report group-level means, standard deviations, and sample sizes for continuous outcomes, as well as event counts and denominators for dichotomous outcomes.

## 5. Conclusions

The meta-analysis did not demonstrate a statistically significant benefit of adjunctive n-3 PUFAs for PANSS total scores, clinical response, all-cause discontinuation, or discontinuation due to adverse events in adults with schizophrenia. However, substantial variation in formulation, dose, duration, population, and concomitant antipsychotic treatment, together with small samples and wide confidence intervals, limits certainty and precludes conclusions about dose–response, formulation-specific effects, or responsive subgroups. Short-term tolerability appeared comparable to placebo, but current evidence does not support routine n-3 PUFA supplementation solely for psychotic symptom reduction. Biomarker- and genotype-informed trials using standardized interventions are needed before a specific role in clinical practice can be defined.

## Figures and Tables

**Table 1:** Eligibility criteria.

Inclusion Criteria	Exclusion Criteria
Participants aged ≥ 18 years. Confirmed diagnosis of schizophrenia according to DSM-IV, DSM-5, DSM-5-TR, or ICD-10 criteria. Concomitant use of typical or atypical antipsychotics as standard treatment (e.g., chlorpromazine, fluphenazine, haloperidol, loxapine, molindone, perphenazine, pimozide, thioridazine, thiothixene, trifluoperazine, aripiprazole, asenapine, brexpiprazole, cariprazine, clozapine, iloperidone, lurasidone, olanzapine, paliperidone, quetiapine, risperidone, and ziprasidone). n-3 PUFA supplementation, regardless of dose, formulation, or duration. Studies conducted in outpatient or inpatient settings.	Pregnancy or lactation. Severe medical comorbidities (e.g., advanced hepatic or renal disease, uncontrolled epilepsy, and severe intellectual disability). Known contraindication to n-3 PUFA supplementation (e.g., allergy, bleeding disorders, use of anticoagulants, or other conditions associated with increased bleeding risk). Quasi-experimental or observational studies.

**Table 2:** Description of the clinical trials included in the systematic review.

Main Author and Year of Publication	Location	Setting	Design	Study Objectives	Sociodemographic and Clinical Variables	Population	Group Treatment Intervention	Treatment in a Control Group	Monitoring Time and Outcome Measurement Times
Qiao et al. 2020 [17].	Shanghai Mental Health Center, School of Medicine, Shanghai Jiao Tong University, Shanghai, China.	Inpatient setting.	Randomized, double-blind, placebo-controlled parallel clinical trial.	To explore the impact of fish oil (n-3 PUFAs) on hostility and psychopathology in patients with acute schizophrenia and violent behavior.	Men/Women: 21/11 (n-3 PUFA) vs. 14/21 (placebo); significant gender difference (*p* = 0.036). Age: 34.09 ± 11.01 vs. 34.03 ± 10.38. Age at first episode: 21.44 ± 6.55 vs. 22.03 ± 7.27. Duration of illness: 12.43 ± 9.92 vs. 11.87 ± 8.11. Baseline MOAS: 14.13 ± 5.63 vs. 14.11 ± 6.72. Baseline hostility (PANSS item): 4.47 ± 1.54 vs. 4.29 ± 1.45. Concomitant medications: clozapine (6 vs. 6) and olanzapine (14 vs. 21).	Schizophrenia (ICD-10) in hospitalized patients with violent behavior. No significant differences between groups were observed in age, age at first episode, duration of illness, baseline MOAS, or baseline hostility; however, sex distribution differed significantly (*p* = 0.036).	Daily fish-oil supplementation containing 540 mg EPA (20:5 n-3) and 360 mg DHA (22:6 n-3) (GNC, Pittsburgh, PA, USA), administered once daily by nursing staff. Plasma EPA and DHA were measured at baseline and week 8.	Placebo: vitamin E capsule 10 mg once daily; identical in color, shape, and texture; both clinical staff and participants were blinded.	Assessments at baseline (week 0), week 4, and week 8 (intervention duration: 8 weeks).
Qiao et al. 2018 [18].	Shanghai Mental Health Center, Shanghai Jiao Tong University School of Medicine, Shanghai, China.	Inpatient setting.	Randomized, double-blind, placebo-controlled trial.	To examine whether daily fish-oil supplementation (540 mg EPA [20:5 n-3] plus 360 mg DHA [22:6 n-3]) reduced symptoms and violent behavior in patients with schizophrenia.	No baseline differences between groups in age (31.98 ± 10.16 years) or sex (n-3 PUFA: 17 men/11 women; placebo: 13 men/9 women). Age at first episode (21.08 ± 7.02 years) and duration of illness (10.80 ± 8.45 years). No baseline differences in CGI, PANSS, or MOAS.	Fifty hospitalized patients with an ICD-10 diagnosis of schizophrenia and MOAS > 4.	Fish oil 540 mg EPA (20:5 n-3) + 360 mg DHA (22:6 n-3) per day as an adjunct to antipsychotic treatment.	Control group treatment: vitamin E 10 mg/day (similar capsule) used as placebo, administered as adjunctive treatment to antipsychotics.	Assessments at baseline and at weeks 4, 8, and 12 for PANSS, MOAS, and CGI. Plasma DHA (22:6 n-3)/EPA (20:5 n-3) levels measured at baseline and at the end of the intervention.
Bošković et al. 2016 [19].	Slovenia: Faculty of Pharmacy, University of Ljubljana (Ljubljana); Department of Psychiatry, University Clinical Centre Maribor (Maribor); University Psychiatric Clinic Ljubljana (Ljubljana).	Outpatient setting.	Randomized, double-blind, placebo-controlled trial with four arms (placebo; vitamin E; EPUFAs; vitamin E + EPUFAs).	To evaluate the effects of supplementation with essential polyunsaturated fatty acids (EPUFAs) and vitamin E in patients with schizophrenia treated with haloperidol, focusing on biomarkers of oxidative stress, neurochemistry, psychopathology, and extrapyramidal symptoms.	At baseline, no significant differences between groups in age, sex, smoking status, weight, duration of illness, haloperidol dose, psychopathology (PANSS), or extrapyramidal symptoms.	Diagnosis according to DSM-IV. All participants were treated with haloperidol 4.2–50 mg/week; 14 also received oral haloperidol 2–30 mg/day.	Vitamin E: Apozema^®^ vitamin E 600 IU, (Apomedica Pharmazeutische Produkte GmbH, Graz, Austria) soft-gel capsules, twice daily. EPUFAs (Omega-3 Forte, Dr. Böhm^®^ Apomedica Pharmazeutische Produkte GmbH, Graz, Austria): per capsule EPA (20:5 n-3) 132 mg, DHA (22:6 n-3) 88 mg, α-linolenic acid 94 mg, oleic acid 52 mg; 3 capsules/day (2 in the morning and 1 in the evening after meals). Combination: both regimens described above. All groups continued intramuscular haloperidol decanoate (4.2–50 mg/week); 14 patients additionally received oral haloperidol 2–30 mg/day.	Placebo: hard gelatin capsules containing 500 mg of lactose. All groups continued intramuscular haloperidol decanoate (4.2–50 mg/week); 14 patients additionally received oral haloperidol 2–30 mg/day.	Measurements at baseline and at 4 months; monthly visits for adherence monitoring.
Bentsen et al. 2013 [16].	Patients admitted to psychiatric departments in Norway, at nine Norwegian hospitals (Southern Norway).	Inpatient setting.	Phase IIB–III multicenter, randomized, double-blind, placebo-controlled, fixed-dose clinical trial with a 2 × 2 factorial design (EPA and/or vitamins E + C as adjunctive treatment).	To examine the clinical effect of adding EPA (20:5 n-3) and/or vitamins E + C to antipsychotic treatment, hypothesizing that lower baseline erythrocyte PUFA levels would predict greater benefit; a synergistic effect was postulated, with the combination expected to yield superior outcomes.	Mean age 27.4 years; 64% male; 72% schizophrenia and 20% schizoaffective disorder; median illness duration 4 years; 31% in first hospitalization; all were receiving antipsychotics.	Diagnosis according to DSM-IV based on the previously described criteria; all participants had antipsychotics prescribed after admission.	EPA (ethyl-EPA) 2 g/day: 2 capsules (500 mg each) twice daily (total 4 capsules/day) for 16 weeks. Vitamins (when applicable): vitamin E 91 mg + vitamin C 250 mg per tablet, twice daily (total E 364 mg/day; C 1000 mg/day).	EPA placebo: capsules containing 500 mg paraffin oil; vitamin placebo: dicalcium phosphate. Group 1 = double placebo.	Five visits every 4 weeks; baseline visit conducted within 4 weeks after admission. PANSS assessed at baseline, week 8, and week 16. Adverse events recorded at each visit. Vital signs and biochemical parameters assessed at the first and last visits. Total duration: 16 weeks.
Manteghiy et al. 2008 [20].	Ibn-E-Sina Psychiatric Hospital, Mashhad, Iran.	Inpatient setting.	Prospective, double-blind, placebo-controlled clinical trial.	To retest the hypothesis that n-3 PUFAs provide antipsychotic benefits in schizophrenia, addressing limitations of previous studies, and to evaluate their adjunctive effect to pharmacological treatment (risperidone).	Age: n-3 PUFA 37.38 ± 6.2 years; placebo 39.03 ± 7.12 (*p* = 0.48). Sex: 76 males (39 n-3 PUFA; 37 placebo) and 9 females (3 n-3 PUFA; 6 placebo) (*p* = 0.25). Education (total sample): 15.5% illiterate; 28.6% primary education; 28.6% secondary education; 27.4% high school/university (*p* = 0.38). Family history of schizophrenia: 10 patients (*p* = 0.50). Number of previous hospitalizations: n-3 PUFA 6.35 ± 3.62; placebo 6.62 ± 3.65 (*p* = 0.73). Age at first admission: n-3 PUFA 25.69 ± 10.04; placebo 26.66 ± 7.37 (*p* = 0.61). Duration of illness (years): n-3 PUFA 14.15 ± 8.3; placebo 15.76 ± 8.67 (*p* = 0.39).	Diagnosis: schizophrenia. Diagnostic criteria: DSM-IV-TR, independently confirmed by two psychiatrists.	Risperidone 2–8 mg/day + n-3 PUFA for 6 weeks. Capsules were initiated at 1 capsule/day on day 1 and increased to 3 capsules/day by day 3 (same regimen maintained for 6 weeks). Each “1 g capsule” of n-3 PUFA reportedly contained 2000 mg of fish oil, 360 mg EPA (20:5 n-3), and 240 mg DHA (22:6 n-3). Only antipsychotic permitted: risperidone (2–8 mg/day).	Risperidone 2–8 mg/day + placebo with identical appearance and dosing schedule (1 capsule/day on day 1, 3/day from day 3 onward) for 6 weeks. Only antipsychotic permitted: risperidone (2–8 mg/day).	Follow-up duration: 6 weeks. Measurements: PANSS at baseline (week 0), week 3, and week 6. AIMS (extrapyramidal side effects) assessed at weeks 3 and 6.
Emsley et al. 2006 [21].	Stikland Academic Hospital and community psychiatric services in the Cape Town metropolitan area, South Africa.	Outpatient setting.	Randomized, double-blind, parallel-group, placebo-controlled trial.	To compare the efficacy of EPA (20:5 n-3) versus placebo in reducing symptoms of tardive dyskinesia. Secondary objectives included comparing EPA (20:5 n-3) versus placebo on psychotic symptoms and assessing safety and tolerability.	Age (mean ± SD): EPA 42.4 ± 10.3 years; placebo 43.4 ± 10.9; *p* = 0.7. Sex (M: F): EPA 27:12; placebo 24:14; *p* = 0.6. Duration of schizophrenia (years): EPA 16.0 ± 10.5; placebo 16.8 ± 10.4; *p* = 0.7. Duration of TD (years): EPA 5.6 ± 4.4; placebo 6.7 ± 7.4; *p* = 0.4. Baseline total PANSS: EPA 59.2 ± 13.0; placebo 57.5 ± 11.8; *p* = 0.6. Baseline ESRS: parkinsonism EPA 8.8 ± 6.6 vs. placebo 9.1 ± 6.1 (*p* = 0.9); dystonia EPA 0.1 ± 0.4 vs. placebo 0.7 ± 1.7 (*p* < 0.0001); dyskinesia EPA 12.0 ± 4.9 vs. placebo 12.5 ± 5.7 (*p* = 0.4); total EPA 32.7 ± 12.9 vs. placebo 35.3 ± 15.4 (*p* = 0.3). Baseline CGI for TD: EPA 3.5 ± 1.0 vs. placebo 4.1 ± 1.1; *p* = 0.01. Baseline antipsychotic treatment: all participants were receiving conventional antipsychotics; none had received second-generation antipsychotics within the previous 6 weeks. Relevant baseline characteristics: groups were similar in demographics and baseline total PANSS; differences were observed in ESRS-dystonia (higher in placebo) and CGI for TD (higher in placebo).	Schizophrenia or schizoaffective disorder with established tardive dyskinesia (DSM-IV criteria). Diagnostic criteria: DSM-IV for both conditions (TD and psychotic disorder).	Ethyl-EPA 2 g/day (2 × 500 mg twice daily; identical capsules), added to the ongoing fixed antipsychotic treatment for 12 weeks. Baseline antipsychotic treatment: all participants were receiving a conventional antipsychotic at a fixed dose (no second-generation antipsychotics in the previous 6 weeks). Concomitant medications permitted: anticholinergics for emergent extrapyramidal symptoms; anxiolytics/hypnotics for emergent insomnia or anxiety; and medications for existing or intercurrent physical conditions.	Placebo (medicinal liquid paraffin BP) 2 g/day in identical capsules, added to the ongoing fixed antipsychotic treatment for 12 weeks. Baseline antipsychotic treatment: all participants were receiving a conventional antipsychotic at a fixed dose (no second-generation antipsychotics in the previous 6 weeks). Concomitant medications permitted: anticholinergics for emergent extrapyramidal symptoms; anxiolytics/hypnotics for emergent insomnia or anxiety; and medication for existing or intercurrent physical conditions.	Duration: 12 weeks. Assessments at baseline and at 3-week intervals using ESRS, CGI for TD, and PANSS.
Peet et al. 2002 [22].	Nine centers in the United Kingdom (E-E Multicentre Study Group). All patients were outpatients under psychiatric follow-up.	Outpatient setting.	Randomized, double-blind, placebo-controlled trial.	To evaluate the effects of ethyl-eicosapentaenoate on persistent symptoms in schizophrenia among patients maintained on clozapine, newer atypical antipsychotics (olanzapine, risperidone, or quetiapine), or typical antipsychotics. The primary outcome was the change from baseline to week 12 in total PANSS score and subscales. Additional assessments included MADRS and side-effect scales, as well as erythrocyte membrane fatty acids and triglycerides (in the clozapine subgroup).	Age (mean; range, years): Placebo 39 (22–61); 1 g 38 (20–60); 2 g 34 (20–62); 4 g 37 (20–56). Sex: male 65%, 66%, 71%, 63%; female 35%, 34%, 29%, 37% (placebo, 1 g, 2 g, 4 g, respectively). Total PANSS (mean; range): 78 (51–132); 75 (50–96); 83 (50–124); 79 (55–109). MADRS (mean; range): 17 (0–49); 15 (0–28); 14 (0–29); 17 (1–44).	Diagnosis: DSM-IV schizophrenia in outpatients with persistent symptoms. Diagnostic criteria: DSM-IV; entry threshold total PANSS ≥ 50 and positive subscale ≥ 15.	Oral E-E (ethyl-EPA) 1 g/day, 2 g/day, or 4 g/day for 12 weeks, administered as eight capsules/day (4 in the morning and 4 in the evening; each capsule 500 mg), added to the unchanged baseline antipsychotic treatment. Baseline antipsychotic treatment maintained without change in drug or dose: clozapine; newer atypical antipsychotics (olanzapine, risperidone, or quetiapine); or typical antipsychotics.	Placebo (medicinal liquid paraffin BP) 4 g/day in identical capsules, administered using the same schedule (4 capsules in the morning and 4 in the evening), added to the unchanged baseline antipsychotic treatment for 12 weeks. Baseline antipsychotic treatment maintained without change in drug or dose: clozapine; newer atypical antipsychotics (olanzapine, risperidone, or quetiapine); or typical antipsychotics.	12 weeks; assessments at baseline and at weeks 4, 8, and 12.
Emsley et al. 2002 [23].	Departments of Psychiatry and Chemical Pathology, University of Stellenbosch; Tygerberg/Cape Town, South Africa.	Outpatient setting.	Randomized, parallel-group, double-blind, placebo-controlled trial.	To investigate the efficacy and tolerability of ethyl-eicosapentaenoate (E-EPA) as adjunctive treatment in chronic, severe schizophrenia.	Age (mean ± SD): E-EPA 46.2 ± 10.6 years; placebo 43.6 ± 13.9. Duration of illness (years, mean ± SD): E-EPA 23.1 ± 8.5; placebo 22.2 ± 12.4. Antipsychotic dose in chlorpromazine equivalents (mg/day, mean ± SD): E-EPA 1011 ± 532; placebo 931 ± 652. Baseline antipsychotic type: 9 subjects in each group were receiving clozapine; the remainder were receiving conventional antipsychotics. Dietary n-3 PUFA intake: low (EPA 0.56–1.13 g/week), with no differences between groups; all participants followed a balanced diet during the trial. Baseline demographic and clinical variables were similar between groups.	Diagnosis: DSM-IV schizophrenia with persistent symptoms despite ≥6 months of fixed-dose antipsychotic treatment. Diagnostic criteria: DSM-IV; entry threshold total PANSS > 50.	E-EPA 3 g/day in capsules (three 500 mg capsules twice daily; manufacturer: Laxdale Ltd., Stirling, Scotland) for 12 weeks, added to the ongoing antipsychotic treatment. Antipsychotic treatment remained unchanged in all patients (clozapine or conventional antipsychotics). Additional medication was limited to occasional analgesics for headache and lorazepam for insomnia.	Placebo 3 g/day (medicinal liquid paraffin BP) in identical capsules for 12 weeks, added to the ongoing antipsychotic treatment. Antipsychotic treatment remained unchanged in all patients (clozapine or conventional antipsychotics). Additional medication was limited to occasional analgesics for headache and lorazepam for insomnia.	Duration: 12 weeks; assessments at baseline and at weeks 3, 6, 9, and 12.
Peet et al. 2001 [24].	Academic Department of Psychiatry, Northern General Hospital, The Longley Centre, Sheffield, UK.	Not specified.	Randomized, double-blind, placebo-controlled pilot trials.	To explore the effects of n-3 fatty acids in schizophrenia.	Not reported.	Formal diagnostic parameters were not reported.	EPA (20:5 n-3) added to stable antipsychotic treatment for 3 months.	DHA (22:6 n-3) and placebo (both administered as co-treatment with stable antipsychotics).	3 months.
Fenton et al. 2001 [25].	National Institute of Mental Health (NIMH), Sheppard and Enoch Pratt Hospital (Towson, MD, USA), National Institute on Alcohol Abuse and Alcoholism (Rockville, MD, USA), and Stanley Foundation Research Programs (Bethesda, MD, USA).	Not specified.	Randomized, double-blind, placebo-controlled trial.	To determine whether supplementation with 3 g/day of EPA (ethyl-eicosapentaenoate) as augmentation to neuroleptic treatment improves symptoms and cognition in schizophrenia or schizoaffective disorder.	Mean age 40 (SD 10) years; 61% male (53/87); 80% single (70/87); 84% Caucasian (73/87); 91% high school graduates (79/87); diagnosis: 70% schizophrenia (61/87) and 30% schizoaffective disorder (26/87); age at onset 20.8 (SD 6.3) years; first hospitalization 21.8 (SD 7.6) years; mean number of previous hospitalizations 10.7 (SD 4.1). All but one participant were receiving a neuroleptic; 22% were on two neuroleptics; 39% were taking risperidone/olanzapine/quetiapine; 28% were taking clozapine.	Diagnosis according to DSM-IV (schizophrenia or schizoaffective disorder) with residual symptoms defined according to PANSS thresholds.	EPA (20:5 n-3) 3 g/day (six capsules/day): each capsule contained 4 mg vitamin E and 500 mg ethyl-EPA. Concomitant treatments: augmentation to ongoing neuroleptic treatment; all but one participant were receiving a neuroleptic; 22% were receiving two neuroleptics; 39% were taking risperidone/olanzapine/quetiapine; 28% were taking clozapine. Eight patients required an increase in neuroleptic dose (4 EPA, 4 placebo).	Mineral oil placebo in identical capsules (also containing vitamin E 4 mg). Concomitant treatments: augmentation to ongoing neuroleptic treatment; all but one participant were receiving a neuroleptic; 22% were receiving two neuroleptics; 39% were taking risperidone/olanzapine/quetiapine; 28% were taking clozapine. Eight patients required an increase in neuroleptic dose (4 EPA, 4 placebo).	16 weeks; assessments at baseline and at weeks 1, 2, 4, 8, 12, and 16; cognitive battery administered at baseline and week 16.

ICD-10: International Classification of Diseases, 10th Revision; MOAS: Modified Overt Aggression Scale; PANSS: Positive and Negative Syndrome Scale; DHA: docosahexaenoic acid (22:6 n-3); EPA: eicosapentaenoic acid (20:5 n-3); CGI: Clinical Global Impression; EPUFAs: essential polyunsaturated fatty acids; PUFAs: polyunsaturated fatty acids; DSM-IV: Diagnostic and Statistical Manual of Mental Disorders, Fourth Edition; DSM-IV-TR: Diagnostic and Statistical Manual of Mental Disorders, Fourth Edition, Text Revision; AIMS: Abnormal Involuntary Movement Scale; ESRS: Extrapyramidal Symptom Rating Scale; TD: tardive dyskinesia; E-EPA: ethyl-eicosapentaenoate; E-E: abbreviation used by the authors for E-EPA; NIMH: National Institute of Mental Health; MADRS: Montgomery–Åsberg Depression Rating Scale; SD: standard deviation; GNC: General Nutrition Corporation.

**Table 3:** Description of the clinical trials included in the systematic review (continuation).

Author and Year	Total Participants	Total Patients in the Intervention Group	Total Patients in the Control Group	PANSS (Positive and Negative Syndrome Scale)	BPRS (Brief Psychiatric Rating Scale)	CGI (Clinical Global Impression)	GAF (Global Assessment of Functioning)	Hospitalization	Relapses	Adverse Effects	Mortality	Comments
Qiao et al. 2020 [17].	67 (35 men, 32 women); 47 (70.1%) completed the 8-week intervention.	Week 0 (baseline): Intervention (fish oil) *n* = 32. Week 4: Not reported. Week 8 (end of intervention): A total of *n* = 47 (70.1%) completed the study, without group-specific breakdown.	Week 0 (baseline): Control (vitamin E placebo) *n* = 35. Week 4: Not reported. Week 8 (end of intervention): A total of *n* = 47 (70.1%) completed the study, without group-specific breakdown.	Baseline: 91.53 ± 16.75 (n-3 PUFA) vs. 88.40 ± 13.80 (placebo). Week 4: 75.53 ± 16.24 vs. 65.44 ± 17.41. Week 8: 67.38 ± 16.54 vs. 59.91 ± 18.67. Repeated-measures ANOVA: *p* < 0.01; time × group interaction F = 0.19. Positive subscale Baseline: 26.50 ± 6.38 vs. 27.09 ± 5.01. Week 4: 19.90 ± 5.62 vs. 17.94 ± 5.93. Week 8: 17.17 ± 5.52 vs. 16.00 ± 6.05. *p* < 0.01. Negative subscale Baseline: 20.84 ± 7.63 vs. 17.91 ± 6.86. Week 4: 18.37 ± 6.99 vs. 14.97 ± 7.43. Week 8: 16.63 ± 6.74 vs. 14.65 ± 7.57. *p* < 0.01. Hostility (PANSS item) Baseline: 4.47 ± 1.54 vs. 4.29 ± 1.45. Week 4: 3.07 ± 1.39 vs. 2.72 ± 1.14. Week 8: 2.38 ± 1.06 vs. 2.42 ± 1.10. *p* < 0.01. Positive, negative, and total PANSS scores decreased in both groups at weeks 4 and 8, with no between-group differences; after adjusting for gender, results remained nonsignificant (*p* = 0.524, 0.353, and 0.165 for positive, negative, and total scores, respectively).	Not reported.	Not reported.	Not reported.	Not reported.	Not reported.	Not reported.	Not reported.	Positive, negative, and total PANSS scores decreased in both groups at weeks 4 and 8, with no differences between groups; after adjustment for gender, the results remained unchanged (*p* = 0.524, 0.353, and 0.165 for positive, negative, and total scores, respectively).
Qiao et al. 2018 [18].	50 participants.	28 participants.	22 participants.	Baseline—Positive: n-3 PUFA 25.86 ± 5.95 vs. placebo 27.00 ± 4.46; t = −0.75; *p* = 0.46. Baseline—Negative: 19.43 ± 8.17 vs. 19.32 ± 7.66; t = 0.05; *p* = 0.96. Baseline—General: 42.46 ± 9.59 vs. 43.45 ± 8.62; t = −0.38; *p* = 0.71. Baseline—Total: 87.75 ± 19.30 vs. 89.77 ± 14.37; t = −0.41; *p* = 0.68. Week 4—Total (*n* = 26 vs. 19): 72.00 ± 19.04 vs. 70.11 ± 15.27; t = 0.36; *p* = 0.72. Week 8—Total (*n* = 21 vs. 14): 62.86 ± 18.52 vs. 61.64 ± 17.16; t = 0.20; *p* = 0.85. Week 12—Total (*n* = 17 vs. 9): 61.65 ± 18.52 vs. 63.00 ± 17.03; t = −0.18; *p* = 0.86. PANSS decreased at weeks 4, 8, and 12 compared with baseline (*p* < 0.01), with no differences between groups.	Not reported.	Baseline—Severity (CGI-SI): category 4: n-3 PUFA 2/placebo 1 (total 3); category 5: 10/6 (total 16); category 6: 16/14 (total 30); category 7: 0/1 (total 1). Between-group difference: *p* = 0.62. Course over time: CGI scores decreased at weeks 4, 8, and 12 compared with baseline (*p* < 0.01), with no differences between groups.	Not reported.	Not reported.	Not reported.	Not reported.	Not reported.	Patients with schizophrenia and violent behavior who received fish oil containing 540 mg EPA (20:5 n-3) and 360 mg DHA (22:6 n-3) showed reduced violence; however, positive and negative symptoms did not improve more than with placebo.
Bošković et al. 2016 [19].	52 participants were included; 34 completed supplementation and were analyzed. Mean adherence 92% (SD 13%).	Across the three active arms: 23 (Vitamin E *n* = 5; EPUFAs *n* = 9; Vitamin E + EPUFAs *n* = 9).	11 (placebo).	Baseline values (median [range]) and between-group comparison at baseline: Total sample (*n* = 34): 56 (34–99). Placebo (*n* = 11): 56 (35–84). Vitamin E (*n* = 5): 44 (34–65). EPUFAs (*n* = 9): 58 (34–99). Vitamin E + EPUFAs (*n* = 9): 43 (35–90). Between-group *p*-value at baseline: 0.670. During the study, no treatment effect was observed on total PANSS or on positive, negative, and general subscales.	Not reported.	Not reported.	Not reported.	Not reported.	Not reported.	Not reported.	Not reported.	Supplementation with vitamin E and EPUFAs may improve antioxidant defense (particularly the glutathione system) without producing a greater effect on symptom severity.
Bentsen et al. 2013 [16].	104 randomized; 99 in the ITT population received study medication.	Factorial design; ITT sample sizes per group were: G1 double placebo 24, G2 active vitamins 26, G3 active EPA 33, G4 active EPA + vitamins 16. For binary comparisons: active EPA (G3 + G4) = 49 vs. EPA placebo (G1 + G2) = 50; active vitamins (G2 + G4) = 42 vs. vitamin placebo (G1 + G3) = 57.	EPA placebo = 50; vitamin placebo = 57.	Modelled results (LMM) depended on baseline PUFA levels: in low PUFA, EPA alone worsened the course of total PANSS by +12.6 points vs. placebo (95% CI 1.0–24.4; *p* = 0.03; d = 0.29); when vitamins were added to EPA, the effect was neutralized (interaction d = 0.31; *p* = 0.02). In high PUFA, no significant effects were observed.	Not reported.	Not reported.	Not reported.	Not reported.	Not reported.	Adverse events were assessed at each visit using the UKU Side Effect Rating Scale (USERS). Five visits were conducted at 4-week intervals; vital signs and biochemical tests were measured at the first and last visits. The study medications (EPA and/or vitamins) were not associated with the total number of USERS adverse events. Nine of 99 patients experienced serious adverse events in the intention-to-treat analysis and 6 of 74 in the per-protocol analysis; two deaths occurred in groups 3 and 4. No association was found between treatment allocation and the number of serious adverse events (Fisher’s exact tests, *p* > 0.48).	Two patients died (groups 3 and 4); no association with treatment allocation was identified.	During an acute episode, adding EPA or vitamins E + C separately does not appear beneficial and may induce psychotic symptoms in patients with low PUFA levels; when combined (EPA + vitamins), they appear to be safe.
Manteghiy et al. 2008 [20].	85 patients (after exclusions and voluntary withdrawals).	43 participants received risperidone + 3 g/day of n-3 PUFA.	44 participants received risperidone + placebo.	n-3 PUFA (mean ± SD) Positive: 50.42 ± 7.56 (week 0); 45.17 ± 9.80 (week 3); 39.40 ± 7.25 (week 6). Negative: 47.97 ± 8.06; 42.85 ± 9.26; 37.20 ± 6.80. General psychopathology: 51.11 ± 8.47; 45.85 ± 10.11; 38.77 ± 7.70. Anergy: 51.88 ± 9.60; 43.65 ± 7.62; 39.28 ± 7.32. Thought disorder: 46.57 ± 6.24; 42.28 ± 7.78; 38.17 ± 5.54. Activation: 52.91 ± 7.33; 48.37 ± 8.36; 44.45 ± 7.26. Suspiciousness: 53.17 ± 8.99; 49.71 ± 8.91; 43.25 ± 6.50. Depression: 49.60 ± 6.60; 49.22 ± 6.73; 45.62 ± 6.58 (change not significant over time, *p* = 0.146). Placebo (mean ± SD) Positive: 49.00 ± 7.68; 44.52 ± 6.01; 39.04 ± 6.50. Negative: 46.83 ± 8.05; 42.61 ± 8.57; 38.61 ± 8.97. General psychopathology: 50.95 ± 9.00; 42.54 ± 7.91; 39.02 ± 8.10. Anergy: 48.80 ± 8.86; 45.64 ± 8.82; 41.28 ± 9.12. Thought disorder: 46.90 ± 5.88; 42.50 ± 5.88; 39.52 ± 5.85. Activation: 50.90 ± 8.22; 46.85 ± 6.74; 44.57 ± 5.95. Suspiciousness: 51.09 ± 8.88; 48.11 ± 7.29; 42.88 ± 6.10. Depression: 51.38 ± 7.28; 46.95 ± 6.73; 44.80 ± 5.88. Between-group comparisons (*t*-test): Week 0: *p* not significant for subscales, although the table reports *p* = 0.025 for positive symptoms (placebo 49.09 ± 7.61 vs. n-3 PUFA 51.02 ± 7.85); the text states no significant PANSS differences at weeks 0, 3, and 6. Week 3: general psychopathology higher in n-3 PUFA (46.46 ± 9.82) vs. placebo (42.60 ± 7.83), *p* = 0.04; other comparisons not significant. Week 6: no significant differences between groups on any subscale.	Not reported.	Not reported.	Not reported.	Not reported.	Not reported.	During the study, six patients developed extrapyramidal side effects and three experienced gastrointestinal side effects; severity was mild, and no participants were excluded. No group-specific breakdown was provided. One patient in the n-3 PUFA group had tardive dyskinesia at baseline, without relevant changes during the study.	Not reported.	n-3 PUFAs did not demonstrate superiority over placebo in reducing positive and negative symptoms of schizophrenia in this trial; the authors suggest that the short study duration limits conclusions and recommend longer clinical trials to clarify the therapeutic role of n-3 PUFAs as adjunctive treatment.
Emsley et al. 2006 [21].	84 randomized; 77 included in the analysis (those with ≥1 post-baseline assessment).	n = 39 (sex distribution M: F 27:12).	n = 38 (sex distribution M: F 24:14).	Baseline: EPA 59.2 ± 13.0 vs. placebo 57.5 ± 11.8; *p* = 0.6. Baseline–final change: no significant between-group differences (*p* = 0.9). Responders (≥20% reduction in total PANSS): EPA 1 (2.5%) vs. placebo 2 (5%); *p* = 0.5. Follow-up PANSS mean ± SD values over time were not reported; only analyses and response rates were provided.	Not reported.	“CGI for TD” was used. Baseline: EPA 3.5 ± 1.0 vs. placebo 4.1 ± 1.1 (*p* = 0.01). The baseline–week 12 change was defined as a secondary outcome, but no numerical comparative results between groups are reported in the text shown.	Not reported.	Not reported.	Not reported.	Two discontinuations due to adverse events occurred in the placebo group (congestive heart failure; epistaxis). Overall rates and group-specific breakdowns were not reported.	Not reported.	The trial did not demonstrate a clear antidyskinetic effect of ethyl-EPA 2 g/day on the primary outcome; mixed-model analysis suggested a modest and transient benefit (up to 6 weeks), possibly greater in cases of shorter-duration tardive dyskinesia. No antipsychotic effect was observed.
Peet et al. 2002 [22].	122 randomized; 115 with ≥1 post-baseline assessment (ITT); 109 completed 12 weeks.	Trial with three active doses. In the ITT population: 1 g *n* = 29, 2 g *n* = 28, 4 g *n* = 27 (total intervention = 84).	Placebo *n* = 31 (ITT).	Patients receiving typical antipsychotics Total PANSS (mean)—Baseline/Final/Change (%): Placebo: 75.0 → 58.4, −16.6 (−22.1%), *p* change = 0.002. 1 g: 73.0 → 63.4, −9.6 (−13.2%), *p* = 0.02. 2 g: 74.8 → 62.4, −12.4 (−16.6%), *p* = 0.05. 4 g: 80.8 → 66.7, −14.1 (−17.5%), *p* = 0.002. Differences vs. placebo: not significant for total and subscales. Patients receiving newer atypical antipsychotics Placebo: 80.3 → 61.3, −19.0 (−23.7%), *p* < 0.001. 1 g: 73.3 → 59.5, −13.8 (−18.8%), *p* = 0.0008. 2 g: 84.7 → 64.9, −19.9 (−23.5%), *p* = 0.0004. 4 g: 82.1 → 73.7, −8.4 (−10.2%), ns. Differences vs. placebo: not significant for total PANSS; in the general subscale, the 4 g group performed worse than placebo (*p* = 0.04). Patients receiving clozapine Total PANSS (mean)—Baseline/Final/Change (%): Placebo: 77.3 → 72.7, −4.6 (−6.0%), ns. 1 g: 80.2 → 65.6, −14.7 (−18.3%), *p* = 0.004. 2 g: 86.1 → 63.7, −22.4 (−26.0%), *p* = 0.005. 4 g: 70.3 → 58.8, −11.5 (−16.3%), *p* = 0.04. Differences vs. placebo: the 2 g dose was superior to placebo for total PANSS (*p* = 0.04) and general psychopathology (*p* = 0.03). No significant differences for positive or negative subscales.	Not reported.	Not reported.	Not reported.	Not reported.	Not reported.	New adverse events (≥1 AE): Placebo 52%; 1 g 50%; 2 g 34%; 4 g 59%. Most frequent events: diarrhea (7, 7, 1, 3) and nausea (0, 1, 3, 2). Withdrawals due to AEs: 1 (placebo), 4 (1 g), 2 (2 g), 2 (4 g). No differences were observed between E-E and placebo in treatment-emergent adverse events; gastrointestinal symptoms were mild and transient. No sedation, sleep disturbances, significant weight changes, or movement disorders attributable to E-E were reported. Hematological/biochemical parameters and liver function showed no clinically significant changes. Triglycerides in patients receiving clozapine decreased significantly with 2 g (*p* = 0.02) and 4 g (*p* = 0.02), with no change in the placebo or 1 g groups.	Not reported.	Within-group improvement was observed in most study arms; however, E-E did not demonstrate superiority over placebo in patients receiving typical or newer atypical antipsychotics. In patients receiving clozapine, E-E 2 g/day showed clinical and statistical improvement compared with placebo in total PANSS and general psychopathology.
Emsley et al. 2002 [23].	40 patients randomized.	20 (E-EPA).	20 (placebo).	Primary outcome (total PANSS, % change baseline–12 weeks): E-EPA 12.6 ± 14.0 vs. placebo 3.1 ± 13.3; t = 2.20, *p* = 0.03 (favoring E-EPA). The difference remained significant after ANCOVA adjusting for dietary EPA intake, medication, duration, and sex and reached significance by week 3. PANSS general psychopathology subscale (% change at 12 weeks): significant difference favoring E-EPA (t = 2.08, *p* = 0.04). Additional analysis: PANSS reduction tended to be greater with conventional antipsychotics than with clozapine (mean 17.4 ± 12.1 vs. 6.8 ± 14.6; t = 1.80, *p* = 0.09). Relationship with dyskinesia: after adjusting PANSS change for dyskinesia change (ESRS), the between-group difference was no longer significant (F = 3.08, *p* = 0.09).	Not reported.	Not reported.	Not reported.	Not reported.	Not reported.	Serious adverse events: one participant in the E-EPA group withdrew due to medication overdose; no other serious adverse events were reported. ESRS: no differences between groups in parkinsonism, dystonia, or akathisia; dyskinesia showed a significantly greater reduction with E-EPA at 12 weeks (t = 2.82, *p* = 0.008).	Not reported.	E-EPA may represent an effective, safe, and well-tolerated adjunctive treatment in chronic schizophrenia; however, the magnitude of its antipsychotic activity remains to be determined. The potential benefit for tardive dyskinesia requires further investigation. The small sample size and the finding that PANSS reduction may be related to dyskinesia reduction limit the generalizability of the results.
Peet et al. 2001 [24].	45 patients.	Not reported.	Not reported.	Improvement with EPA (% change in total PANSS) was statistically superior to DHA (22:6 n-3) and placebo; EPA was also superior to DHA (22:6 n-3) for positive symptoms (repeated-measures ANOVA). Mean ± SD values were not reported.	Not reported.	Not reported.	Not reported.	Not reported.	Not reported.	Not reported.	Not reported.	The authors conclude that EPA may represent a novel therapeutic approach in schizophrenia, supporting its evaluation in large-scale controlled trials.
Fenton et al. 2001 [25].	90 enrolled; completed: *n* = 75.	*n* = 43 (randomized/ITT); 37 completers.	*n* = 44 (randomized/ITT); 38 completers.	EPA: Week 0 74 ± 16 → Week 16 69 ± 16. Placebo: Week 0 76 ± 18 → Week 16 70 ± 18. Repeated-measures analysis: significant time effect, without a time × group interaction.	Not reported.	EPA: week 16, 3.5 ± 0.7. Placebo: week 16, 3.5 ± 0.7. Repeated-measures analysis: significant time effect, with no time × group interaction.	Not reported.	Not reported.	Not reported.	Events ≥ 5% in the EPA group: upper respiratory infection 19% (8/43) and diarrhea 19% (8/43).	Not reported.	PANSS/CGI: improvement over time was observed in both groups, with no differences between EPA and placebo.

PANSS: Positive and Negative Syndrome Scale; BPRS: Brief Psychiatric Rating Scale; CGI: Clinical Global Impression; CGI-SI: Clinical Global Impression–Severity Index; GAF: Global Assessment of Functioning; ITT: intention-to-treat; LMM: linear mixed model; PUFA: polyunsaturated fatty acid; UKU: UKU Side Effect Rating Scale; USERS: UKU Side Effect Rating Scale (abbreviation used by the authors); ANCOVA: analysis of covariance; ESRS: Extrapyramidal Symptom Rating Scale; TD: tardive dyskinesia; EPA: eicosapentaenoic acid (20:5 n-3); DHA: docosahexaenoic acid (22:6 n-3); SD: standard deviation; ns: not significant; AE: adverse event.

## Data Availability

No new data were created or analyzed in this study. Data sharing is not applicable to this article.

## Supplementary Materials

The following supporting information can be downloaded at https://www.mdpi.com/article/10.3390/brainsci16090928/s1; File S1: PRISMA 2020 Main Checklist; Table S1: Complete PubMed/MEDLINE electronic search strategy used for the systematic review.

## Abbreviations

The following abbreviations are used in this manuscript:

AE	Adverse events
AIMS	Abnormal Involuntary Movement Scale
ANCOVA	Analysis of covariance
BPRS	Brief Psychiatric Rating Scale
CGI	Clinical Global Impression
CGI-SI	Clinical Global Impression–Severity Index
CI	Confidence interval
ICD-10	International Classification of Diseases, 10th Revision
DHA	Docosahexaenoic acid (22:6 n-3)
DSM-IV	Diagnostic and Statistical Manual of Mental Disorders, Fourth Edition
DSM-IV-TR	Diagnostic and Statistical Manual of Mental Disorders, Fourth Edition, Text Revision
E-E	Abbreviation used by the authors for E-EPA
E-EPA	Ethyl-eicosapentaenoate
EPA	Eicosapentaenoic acid (20:5 n-3)
EPUFAs	Essential polyunsaturated fatty acids
ESRS	Extrapyramidal Symptom Rating Scale
GAF	Global Assessment of Functioning
GNC	General Nutrition Corporation
ITT	Intention-to-treat
LMM	Linear mixed model
MADRS	Montgomery–Åsberg Depression Rating Scale
NIMH	National Institute of Mental Health
NS	Not significant
PANSS	Positive and Negative Syndrome Scale
PUFA	Polyunsaturated fatty acid
SD	Standard deviation
TD	Tardive dyskinesia
UKU	UKU Side Effect Rating Scale
USERS	UKU Side Effect Rating Scale (abbreviation used by the authors)
