# Supplementary material for: Omega-3 Polyunsaturated Fatty Acids as Adjunctive Therapy to Antipsychotic Treatment in Adults with Schizophrenia: A Systematic Review and Meta-Analysis
Source: Brain Sci. 2026 Aug 31;16(9):928. doi: 10.3390/brainsci16090928 (PMC13604507; doi:10.3390/brainsci16090928)
Supplement: Supplementary file 1 [file brainsci-16-00928-s001.zip › Table S1 Complete PubMed MEDLINE electronic search strategy used for the systematic review.pdf]

Supplementary Table S1. Complete PubMed/MEDLINE electronic search strategy used for the systematic review.

Database	PubMed/MEDLINE
Date searched	June 2026
Records retrieved	40
Complete electronic search strategy	(<br>"Polyunsaturated"[Title/Abstract]<br>OR "Polyunsaturated fatty acids"[Title/Abstract]<br>OR "PUFA"[Title/Abstract]<br>OR "Fatty Acid"[Title/Abstract]<br>OR "Omega-3 Fatty Acid*"[Title/Abstract]<br>OR "Omega 3 Fatty Acid*"[Title/Abstract]<br>OR "Docosahexaenoic Acid"[Title/Abstract]<br>OR "DHA"[Title/Abstract]<br>OR "Docosahexaenoic"[Title/Abstract]<br>OR "Eicosapentaenoic Acid*"[Title/Abstract]<br>OR "EPA"[Title/Abstract]<br>OR "Alpha Linolenic Acid"[Title/Abstract]<br>)<br><br>AND<br><br>(<br>"Schizophrenia"[Title/Abstract]<br>OR "Schizophrenias"[Title/Abstract]<br>OR "Schizophrenic Disorder*"[Title/Abstract]<br>OR "Schizophrenic"[Title/Abstract]<br>)<br><br>AND<br><br>(<br>Chlorpromazine[Title/Abstract]<br>OR Fluphenazine[Title/Abstract]<br>OR Haloperidol[Title/Abstract]<br>OR Loxapine[Title/Abstract]<br>OR Molindone[Title/Abstract]<br>OR Perphenazine[Title/Abstract]<br>OR Pimozide[Title/Abstract]<br>OR Thioridazine[Title/Abstract]<br>OR Thiothixene[Title/Abstract]<br>OR Trifluoperazine[Title/Abstract]<br>OR Aripiprazole[Title/Abstract]<br>OR Asenapine[Title/Abstract]<br>OR Brexpiprazole[Title/Abstract]

	OR Cariprazine[Title/Abstract]<br>OR Clozapine[Title/Abstract]<br>OR Iloperidone[Title/Abstract]<br>OR Lurasidone[Title/Abstract]<br>OR Olanzapine[Title/Abstract]<br>OR Paliperidone[Title/Abstract]<br>OR Quetiapine[Title/Abstract]<br>OR Risperidone[Title/Abstract]<br>OR Ziprasidone[Title/Abstract]<br>OR "Antipsychotic*"[Title/Abstract]<br>OR "Typical antipsychotics"[Title/Abstract]<br>OR "first-generation antipsychotics"[Title/Abstract]<br>OR "Atypical antipsychotics"[Title/Abstract]<br>OR "second-generation antipsychotics"[Title/Abstract]<br>)<br><br>AND<br><br>(<br>"Clinical Trial"[Title/Abstract]<br>OR "Trial"[Title/Abstract]<br>OR "Randomized Controlled Trial"[Title/Abstract]<br>OR "Randomized"[Title/Abstract]<br>)

Note. The PubMed/MEDLINE search strategy is presented as the reference strategy. Equivalent search strategies were adapted for the remaining databases by modifying the syntax while preserving the same conceptual framework.

Abbreviations. DHA, docosahexaenoic acid; EPA, eicosapentaenoic acid; PUFA, polyunsaturated fatty acid.
